# Injection-Less Conductance Method for Vascular Sizing

**DOI:** 10.3389/fphys.2018.00371

**Published:** 2018-04-12

**Authors:** Ali E. Dabiri, Ghassan S. Kassab

**Affiliations:** ^1^3DT Holdings, San Diego, CA, United States; ^2^California Medical Innovations Institute, San Diego, CA, United States

**Keywords:** conductance catheter, artery disease, saline injection, lumen area, vessel profile

## Abstract

Lumen vessel sizing is important for optimization of interventional outcomes for treatment of vascular disease. The objective of this study is to develop an injection-less method to determine the lumen diameter, using multiple frequencies that eliminates the need for saline injections. We utilize the same electrical conductance devices developed for the two-injection method. A mathematical electrical model was devised to estimate the lumen area and diameter of the arteries. *In vitro* experiments were used to validate the method for various lumen diameters with both 5-5-5 (peripheral) and 2-2-2 (coronary) spacing conductance guidewires. The majority of 11 vessel data fall within one standard deviation and all the data fall within two standard deviations. The results indicate that the two-frequency model can reasonably predict the lumen diameter in an *in-vitro* test set-up. Our findings show that this approach can potentially translate to *in vivo* which would enable pull-back to reconstruct the lumen area profile of the vessel.

## Introduction

The clinical significance of accurate sizing of an artery for percutaneous treatment has been well established by numerous randomized clinical trials (Katzen et al., 1991; Arko et al., 1998; Lee et al., 1998). Under-sizing causes an increase in restenosis rates and oversizing may cause dissection, perforation, or acute vessel closure. The value of optimal sizing during percutaneous transluminal angioplasty (PTA) has been validated in numerous studies using intravascular ultrasound (IVUS). For PTA, IVUS improves procedural results due to optimal balloon sizing leading to significant improvement in luminal dimensions (Katzen et al., 1991; White et al., 1997; Arko et al., 1998; Lee et al., 1998). Despite the utility of IVUS, it is not used routinely in the clinic because of the added time, complexity, subjective interpretation of images, cost, and required training associated with its usage. Angiography (visual estimation or “eye balling” and quantitative angiography, QA), however, is used more routinely but uses a 2D slice projection of a 3D vessel and relies upon edge detection (which assumes a circular vessel). Hence, QA lacks accuracy for sizing because of spatial resolution and irregularity of vessel geometry (i.e., non-circular diseased vessels). Incorrect sizing visual estimation and QA has been shown to lead to suboptimal therapy delivery and diminished patient outcomes (Katzen et al., 1991; Dietz et al., 1992; Di Mario et al., 1992; Keane et al., 1995; White et al., 1997; Arko et al., 1998; Lee et al., 1998). We have performed pre-clinical and clinical studies to validate the functionality of our 0.035′′ LumenRECON (LR) guidewire as a standard workhorse guidewire for vessel navigation and an accurate diagnostic tool for luminal sizing in comparison with other imaging modalities (e.g., QA, IVUS and duplex ultrasound). The lumen sizing has been performed by two-bolus injections of saline solutions with different salinities (normal and half normal) (Kassab et al., 2004, 2005, 2009; Hermiller et al., 2011; Choi et al., 2017; Nair et al., 2018).

The saline injections complicate a standard pullback procedure to determine continuous real-time quantitative measurement of lumen cross sectional area (CSA). Furthermore, the assumption of the injection method is that the saline solution will transiently displace the blood during the injection. This requires good engagement of the introducing catheter to allow a brisk flush (similar to contrast injection for an angiogram). Finally, an additional underlying assumption is that the parallel conductance, *G*_*p*_, is constant over the injections of two different saline solutions. This assumption may be challenged when *G*_*p*_becomes excessively high (e.g., when over 90% of current is lost through the vessel wall and surrounding tissue) (Choi et al., 2017).

Although there are several advantages of the electrical conductance technology given the ease of use in comparison with IVUS (real-time measurements, no need for interpretation, etc.) in a recent first in man (Nair et al., 2018), the latter does not require a saline flush (unlike optical coherence tomography, OCT). Here, a methodology was developed to eliminate the need for saline injections using multiple frequencies to calculate the vessel lumen cross-sectional area. Although similar technique has been attempted to estimate the left ventricular volume (Wei, 2004), they have been based on empirical parameters (as compared to our approach which is entirely physics-based) or have involved the need for parameters that are invasively measured and hence not clinically translational.

The objective of this study is to develop a two-frequency method that eliminates the need for saline solution injections. The basic premise is to vary the electrical admittance by varying energy (i.e., frequency) rather than salinity. We validate this approach in phantoms and in *ex-vivo* vessels. We utilize the same conductance guidewire that has been developed for the two-injections method (5-5-5 spacing for peripheral and 2-2-2 guidewire for coronary vessels). This study provides the foundation for a method to measure the vessel lumen diameter in real-time as well as to allow pullback profiles of vessel lumen with unprecedented accuracy to guide therapy delivery for treatment of vascular disease.

## Methods

Mathematical modeling of this technique is described in the Appendix of this paper. Here, we describe the general method to obtain the lumen and vessel wall tissue conductivity. We also describe the method for the *ex-vivo* study. Finally, both 5-5-5 and 2-2-2 spacing wires were used in the experiments to measure the diameters of peripheral (4–10 mm diameter range) and coronary (2–5 mm diameter range), respectively. The spacing numbers represent the distance between the four electrodes in mm. The larger spacing is associated with the 0.035′′ diameter guidewire for peripheral vessels while the smaller one is associated with the 0.014′′ diameter guidewire for coronary vessels. This is to ensure that the excitation to excitation distances, *d* (18 mm and 9 mm for 5-5-5 and 2-2-2, respectively) is approximately twice the diameter (*d*_*b*_) of the largest vessel of interest (10 and 4 for peripheral and coronary, respectively; i.e., d/*d*_*b*_ ≥ 2) to obey the cylindricity assumptions (Choi et al., 2017).

### Phantom studies

The blood and lumen wall tissue conductivities are needed to estimate the lumen diameter by the injection-less method. The ideal method to determine the blood conductivity in the lumen is in phantoms where the diameter is known and the surrounding tissue conductivity is zero. The blood conductance was estimated using the two-frequency approach. The admittance at various frequencies was calculated by applying the values obtained from equations A8 and A9 (see Appendix) for frequencies of 10 and 100 kHz. The blood conductivities were calculated from the value (system resistance) determined from the two-frequency model (Appendix).

### *Ex-vivo* studies

The objective of the *ex-vivo* study was to estimate the mean and standard deviation (SD) of the difference in vessel diameter as measured by an optical method and the model prediction for different lumen sizes. Eleven *ex-vivo* experiments were performed to validate the model for both 5-5-5 and 2-2-2 guidewires with 0.45% saline solution flowing inside the lumen. The lumen diameters ranged from 1.7 to 8 mm (TR range of 0.16–0.9). An estimate of the vessel wall electrical conductivity is needed to predict the vessel diameter using the two-frequency approach. The bovine carotid artery was placed in de-ionized water bath perfused with the 0.45% saline at room temperature to measure the total impedance of tissue. The lumen diameter and the tissue thickness were measured optically. A two-frequency model was used to convert the measured voltage to the sum conductance of the lumen and the tissue wall. The lumen conductance can be obtained from the known diameter of the lumen and the conductivity of the saline solution which was obtained from the phantom experiments. The tissue conductance can be determined as the total minus the lumen conductance. The tissue electrical conductivity was calculated from the tissue conductance and the cross-sectional area of the tissue. Experiments were performed with bovine carotid artery to estimate the electrical conductivity of the tissue surrounding the artery at room temperature. The artery diameter was measured optically to be about 3.2 mm with the thickness of the vessel wall tissue of about 2.5 mm at no-load state (zero pressure condition). The excitation current was 100 μ*A rms* and the voltages were measured in the frequency range of 10–80 kHz.

The bath is constructed from a Plexiglas box with dimensions of 9H × 25W × 25L cm which contains the vessel and the necessary plastic tubes for the saline solution flow. The bath was filled with the de-ionized water to measure the tissue conductivity. The water fully covered the vessel with 4.5 cm above the height of vessel. The bath width was varied by placing circular tube with various diameters around the vessel to be able to calculate the contribution of parallel conductance to the total conductance as function of bath diameter. In this mode of operation, the bath is filled with 0.1% saline solution which closely represents the *in-vivo* condition.

Once the tissue conductivity is known, it can be used to analyze the mean differences in diameter as measured by the optical method and the model prediction for different lumen sizes as follows. The total conductance can be separated into two components, lumen conductance, *G*_*b*_ and parallel conductance, *G*_*P*_. The following equation holds for the two electrical conductances:

(1) $$G=G_{b}+G_{P}=1/R$$

The lumen diameter can be obtained by combining Equations (A2), (A7), and (1) to yield following relation:

(2) $$d_{b}=\sqrt{\frac{4L+\pi R\sigma_{bIdeal}d_{GW}^{2}}{\pi R\left(\sigma_{bIdeal}+4\sigma_{tIdeal}TR\left(1+TR\right)\right)}}$$

where *d* is the excitation electrode separation distance, *L* is the sensing electrode separation distance, σ_*bIdeal*_ and σ_*tIdeal*_ are the blood and tissue conductivities, respectively; *d*_*GW*_ is the diameter of the guidewire, *R* is the system resistance defined in the two frequency model (see Appendix), *TR* is the ratio of the wall tissue thickness and lumen diameter, and *d*_*b*_ is the lumen diameter. The lumen diameter can be estimated from the total conductance, and the conductivities of the blood and the vessel wall tissue. The blood conductivity is both a function of frequency and lumen diameter. An iterative method was used to calculate the lumen diameter. A two-frequency model was used to determine the *R* resistance of the total system by measuring the voltages at two frequencies.

Series of *ex-vivo* experiments were performed to determine the ratio of lumen conductance to parallel conductance. The tests were performed with the bovine carotid artery immersed in a 0.1% NaCl solution bath to simulate the level of parallel conductance *in vivo*. The width or diameter of the bath around the vessel was varied in four dimensions of 2.1, 4.9, 11.7, and 20.9 mm. The 0.45% saline solution was used to perfuse through the vessel lumen. The guidewire had electrode spacings of 5-5-5 and the applied current was 300 μ*A* rms. At each step, the voltages were measured at two frequencies. The first experiment was performed without any parallel conductance (suspended in air) to calculate the vessel wall tissue conductivity.

The methodology for the determination of vessel diameter using the two-frequency method is an iterative process as follows.

**Figure 1:**
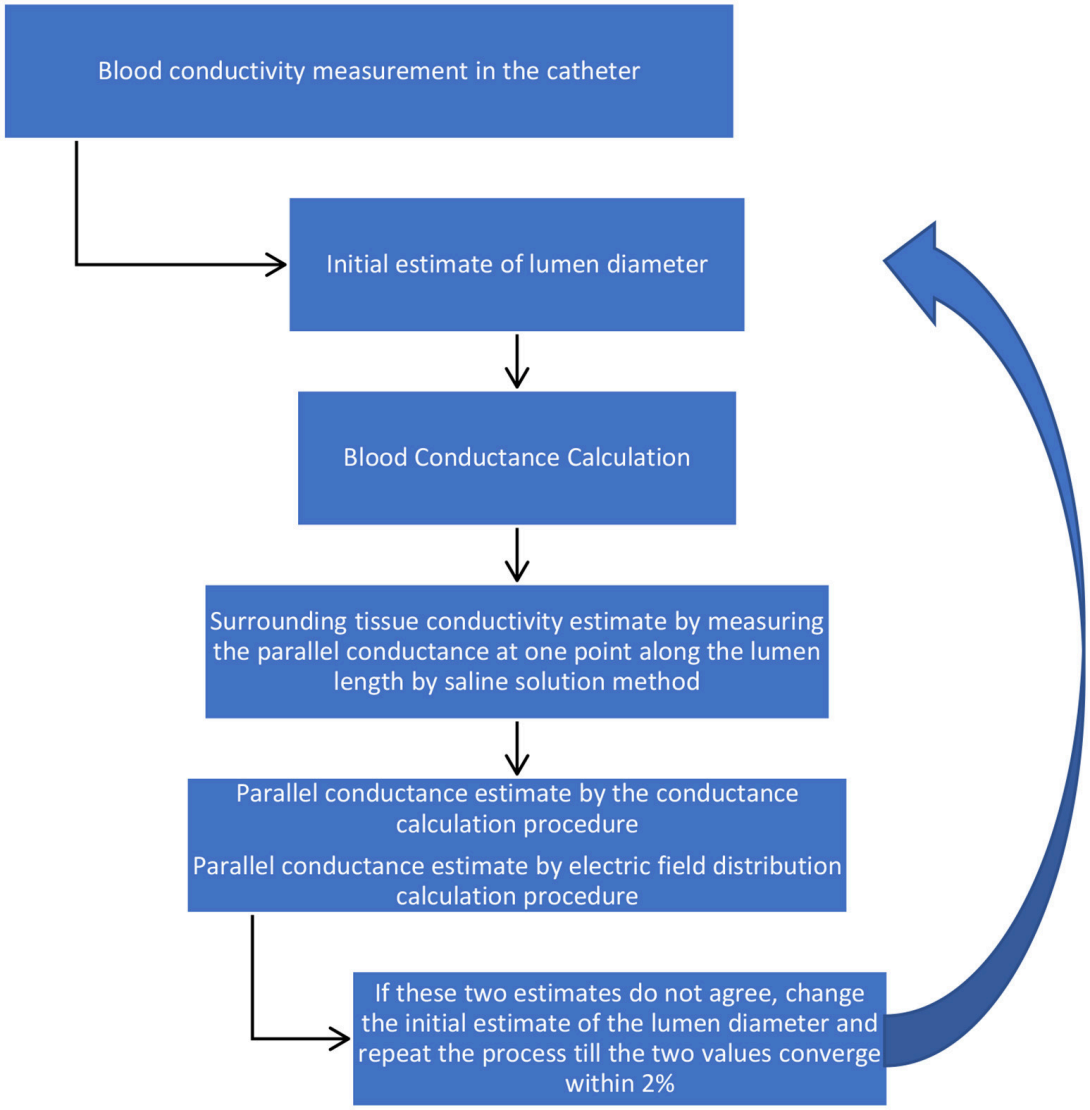
Injection-less method for pull back procedure.

1. Blood conductivity: For *ex-vivo* conditions, the description of blood conductivity measurements in phantoms is described above. Under *in vivo* conditions, the guidewire will be inserted in the standard introducing catheter (typically 5Fr or 6Fr) with some aspiration of subject blood into the catheter to measure blood voltage drops across the detection electrodes at 10 and 20 kHz. The value of voltage differences at these two frequencies are large enough for small lumen diameters. The voltages will be converted to the impedance by dividing the electrical current to the measured voltages. The blood conductance in the catheter will be calculated from Equation (A8, Appendix) which is the inverse of *R*. The blood ideal conductivity in the catheter will be calculated since the diameter of the introducer catheter is known.
2. Total conductance: The sizing guidewire will then be inserted in the lumen of blood vessel to measure blood voltage drops across the detection electrodes at 10 and 20 KHz. The voltages will be converted to the impedance by dividing the electrical current to the measured voltages. The total conductance in the lumen will be calculated from Equation (A8, Appendix) which is the inverse of *R*.
3. Blood conductance: Equation (A2, Appendix) will be applied to determine the blood conductance,*G*_*b*_, using the blood conductivity in the lumen obtained in step 1. This term is only a function of the lumen diameter which is the variable of interest. The blood conductance will be calculated by an initial estimate of the lumen diameter.
4. Parallel conductance: The parallel conductance will be determined from Equation (1) by *G* − *G*_*b*_, which is only function of lumen diameter. This is based on assumption that the tissue wall conductance makes up a portion of the parallel conductance. The parallel conductance will be obtained by the integration of a term from the lumen wall to the surrounding tissue thickness. This term is a product of the tissue conductivity, the electric field at any point within the surrounding tissue thickness and the corresponding annular surface area. The value of the parallel conductance from this calculation is also a function of the lumen diameter since the integration starts from the lumen wall. The parallel conductance has been modeled and discussed in the next section. The model results indicate that there is a fixed ratio of the parallel conductance to total conductance for a specific guidewire, specific blood, specific diameter, specific surrounding tissue conductivity, and specific surrounding tissue thickness. Any resulting error associated with the parallel conductance calculation will result in a similar error in the lumen conductance calculation, which in turn results in an error in the lumen diameter calculation. The error in the lumen diameter is almost half the error in the lumen conductance for small errors since the lumen diameter is proportional to the square root of lumen conductance.
5. Diameter determination: The diameter can be determined by setting the values of the parallel conductance determined by the two procedures to be equal. If this value of the lumen diameter is not the same as the initial estimate, then a new value will be selected and the process will be repeated till the parallel conductance from the two procedures approach each other within 2%. The schematic of the methodology is shown in Figure 1.

### Statistical analysis

The relation between the predicted lumen diameter vs. optically measured were expressed by linear least squares fit and a corresponding correlation coefficient *R*^2^. In a Bland-Altman scatter diagram, we plotted the difference in these two diameters against their means (Bland and Altman, 1986). We assessed the repeatability of the technique by making measurements in duplicates. We calculated the mean and standard deviation of the differences. We used the standard deviation of the differences as the repeatability coefficient (Standards British Standards Institution, 1979).

## Results

### Phantom experiments

The experiments were performed at room temperature with both blood and 0.45% NaCl saline solution in phantoms of various diameters with 5-5-5 and 2-2-2 guidewires from 0.1 to 100 KHz with 100 μ*A* rms current of sinusoidal shape. The measured voltages are shown in Table A1 in Appendix for the 0.45% saline solution for 5-5-5 guidewire at room temperature.

The ideal conductivities have been calculated from the *R* resistance values determined from the two-frequency model. Example of *R* values is shown in Table A2 in Appendix for lumen diameter of 4 mm with 0.45% saline solution at room temperature at different frequencies. The *R* values have been calculated for all combinations of two frequencies. These combinations have been grouped into five categories. In each category, the first frequency is the same while the second frequency was made to vary. It is observed that the value of *R* (inverse of conductance) is relatively constant in each group and decreases with increase of frequency. This results in increase of conductivity with frequency. The ideal conductivities are calculated from *R* values and lumen diameters and are shown in Table 1. The conductivities decrease with diameter and increase with frequency. The same procedure was performed with 2-2-2 guidewire and the ideal conductivities results are shown in Table A3 in Appendix for 0.45% saline solution.

**Table 1:** Calculated ideal conductivity of 0.45% saline solution for various phantom diameters as function of frequency at room temperature for 5-5-5-guidewire.

**Conductivity (σ)**	**Diameter (mm)**				
**(S/m)**	**1.75**	**3**	**4**	**6**	**8**
**FREQ (Hz)**					
10,000	1.7000	1.6000	0.9500	0.8800	0.8000
20,000	2.2000	1.6000	1.0000	0.9000	0.8100
40,000	2.4000	1.6000	1.0300	0.9200	0.8200
60,000	2.6000	1.7000	1.0400	0.9500	0.8300
80,000	2.8000	1.8000	1.0600	0.9500	0.8300

### *Ex-vivo* experiments

Table 2 shows the tissue wall conductivities at various frequencies where the lumen diameter is 3.2 mm with wall thickness of 2.5 mm (TR was calculated to be 0.79). The tests were performed at room temperature with 5-5-5 guidewire with 0.45% saline solution flowing inside the lumen. The results indicate that tissue conductivity is essentially constant in this frequency range.

**Table 2:** Vessel wall tissue conductivities at various frequencies at room temperature for 0.45% saline solution flowing inside the lumen.

	**Diameter (mm)**			
	**σ_*bIdeal*_**	**GL**	**Gt**	**σ_*tIdeal*_**
**Freq (Hz)**	***d*** = **3.2 mm**	**mS**	**mS**	**S/m**
10	1.4	1.75	2.97	0.39
20	1.5	1.88	2.98	0.39
40	1.4	1.75	3.12	0.41
60	1.5	1.88	3.04	0.40
80	1.6	2.01	3.16	0.41

*GL and Gt are the lumen and tissue conductance respectively*.

**Figure 2:**
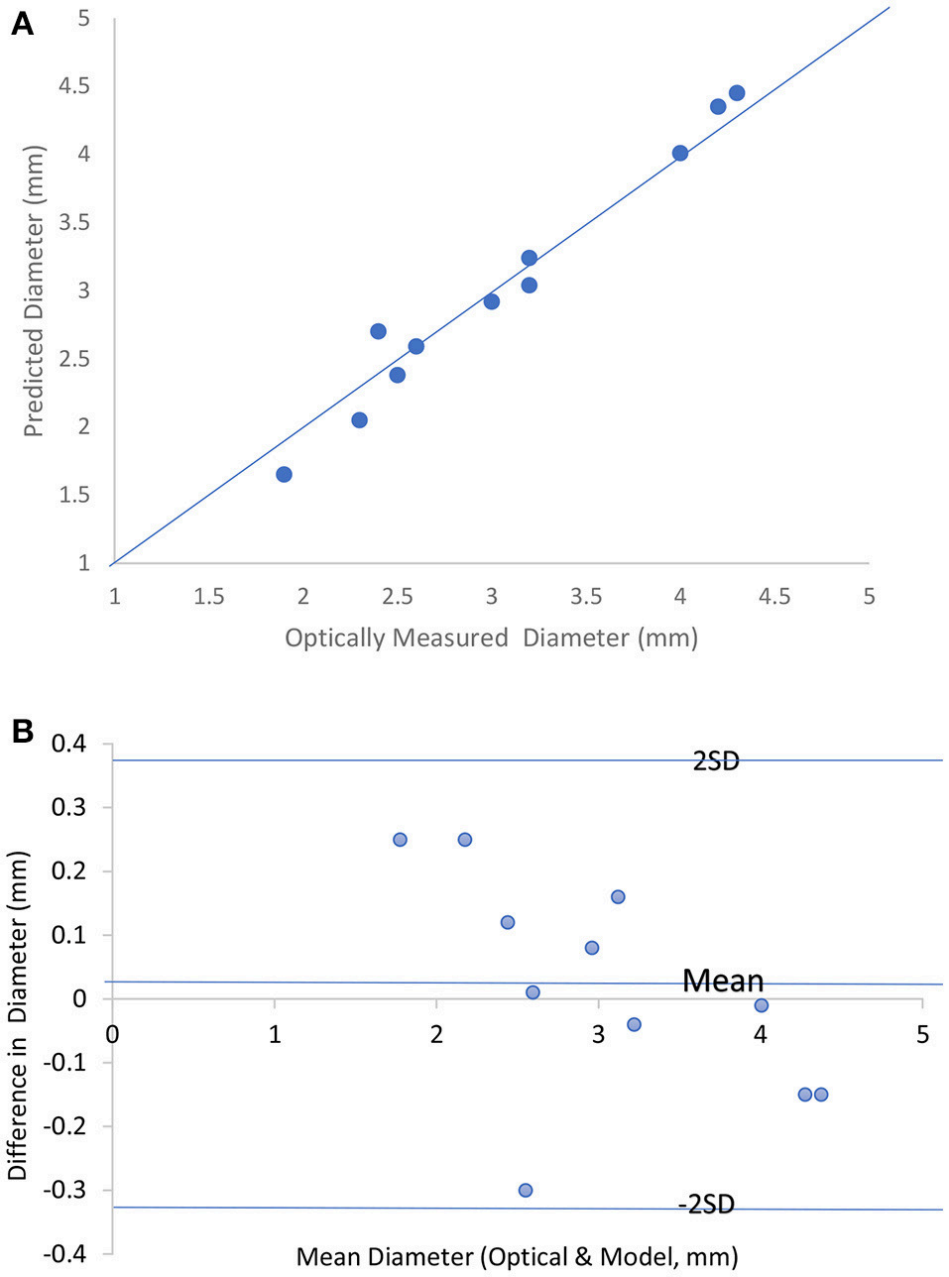
**(A)** Predicted lumen diameter vs. optically measured for the 11 *in vitro* tests with the line of equality. **(B)** Bland-Altman chart for the 11 *in vitro* tests.

Figure 2A shows the predicted lumen diameter vs. optically measured for 11 *ex-vivo* tests with the line of equality. The tissue conductivity for all the tests data analysis which is required as input to the model were set to be 0.4 S/m according to Table 2. The saline solution conductivities were obtained from the conductivities tables by iteration to correspond to the final lumen diameter prediction. The figure compares the diameters obtained by the optical measurement and the model prediction for the 11 experiments. The Bland-Altman plot is shown in Figure 2B. The abscissa of this chart represents average diameter obtained from optical measurement and model prediction for the 11 experiments. The ordinate of this chart represents the difference in these two diameters for the 11 data points. One standard deviation (1SD) was 0.17 mm and the majority of the data (70%) fall within 1SD and all the data fall within 2SDs. The results indicate that the two-frequency model can accurately predict the lumen diameter in *ex-vivo* measurements.

A sensitivity analysis was performed with respect to variation in the tissue thickness. The result indicates that the lumen diameter changes by 1–7% for 20% change in the tissue wall thickness depending on the ratio of the wall thickness to lumen diameter (0.2–0.6). Another sensitivity analysis was performed with respect to variation in tissue conductivity. The result indicates the lumen diameter changes by 2–5% for 20% change in the tissue conductivity depending on the ratio of the wall thickness to lumen diameter (0.2–0.6).

Table 3 shows the variation of total conductance with respect to bath diameter. The parallel conductance increases with the bath diameter since the increase in the total conductance is only due to the bath diameter. Table 4 demonstrates the ratio of bath conductance to the total conductance as function of the bath diameter at different frequencies for the same vessel. The ratio is defined as the ratio of bath conductance at specified bath diameter to the total conductance at the diameter of 20.9 mm (maximum). The table was constructed by subtracting lumen and vessel wall tissue conductance from the total conductance at each specified bath width. Table 4 indicates that about 65% of the total conductance goes to the bath. This value is for very thick vessel wall since TR is about 0.8 (bovine vessel). The parallel conductance increases if TR is reduced. Table 5 shows the results of the variation of ratio of the parallel conductance to the total conductance with respect to TR predicted by the model. For example, this ratio increases to 82% when TR is reduced to 0.3. This corresponds to wall thickness of about 1 mm for this vessel with diameter of 3.2 mm.

**Table 3:** Variation of total conductance with respect to the bath thickness.

**G_total**	**t_bath(mm)**				
**Freq (kHz)**	**0.000**	**2.118**	**4.868**	**11.668**	**20.868**
10	4.6579	6.4886	8.3578	11.3277	12.8155
20	4.6579	6.6399	8.6467	11.9102	13.4538
40	4.7826	6.6144	7.9449	11.6696	12.4735

**Table 4:** Ratio of experimental values of parallel conductance to the total conductance as function of the bath thickness at different frequencies, *TR* = 0.79.

**%(G_bath/G_total(r_bath_MAX))**	**t_bath (mm)**			
**Freq (kHz)**	**2.118**	**4.868**	**11.668**	**20.868**
10	14.29%	28.87%	52.04%	63.65%
20	14.73%	29.65%	53.91%	65.38%
40	14.68%	25.35%	55.21%	61.66%

**Table 5:** Variation of the ratio of parallel conductance to the total conductance with respect to TR, predicted by the model.

		**t_bath(mm)**				
		0.000	2.118	4.868	11.668	20.868
*TR* = 0.1		0.00%	33.94%	47.09%	79.15%	87.66%
*TR* = 0.3		0.00%	28.84%	41.99%	74.05%	82.56%
*TR* = 0.5		0.00%	22.29%	35.44%	67.50%	76.00%
*TR* = 0.8		0.00%	9.72%	22.88%	54.94%	63.44%

**Figure 3:**
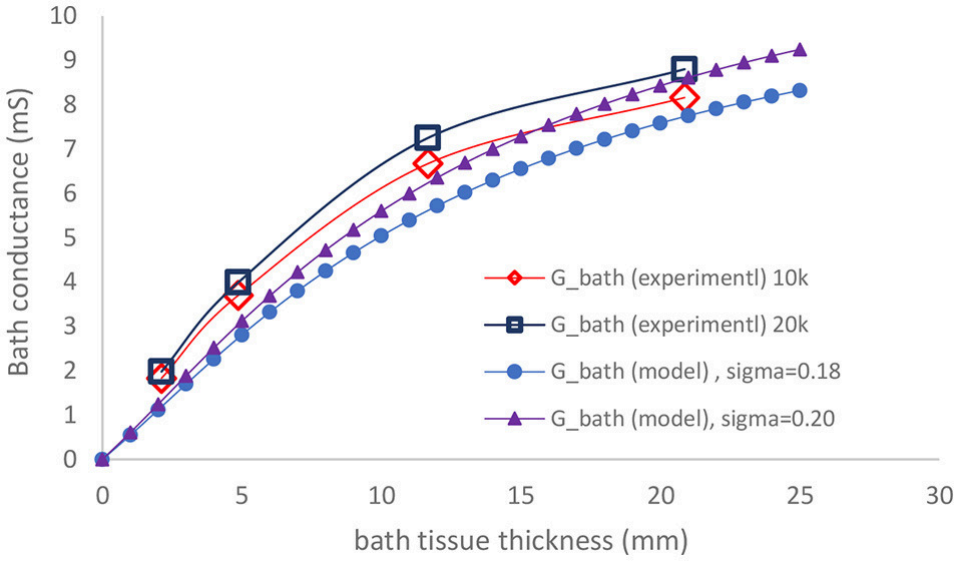
Comparison of measured parallel conductance at 10 and 20 KHz, with model prediction as function of bath thickness.

The experimental results of the parallel conductance (measured for two frequencies of 10 and 20 KHz) and the model predictions for the two bath conductivities (both similar to the conductivity of 0.1% saline solution used in the experiments) are plotted in Figure 3. The agreement is very good for various surrounding tissue thicknesses.

## Discussion

The major finding of this study is that a two-frequency approach can be used to determine the lumen area and hence diameter of *ex-vivo* blood vessels using a capacitive model that requires two physical parameters: tissue conductivity and tissue thickness. A sensitivity analysis was performed to show that the lumen area is not sensitive to tissue conductivity and tissue thickness provided these parameters are in the normal range.

**Figure 4:**
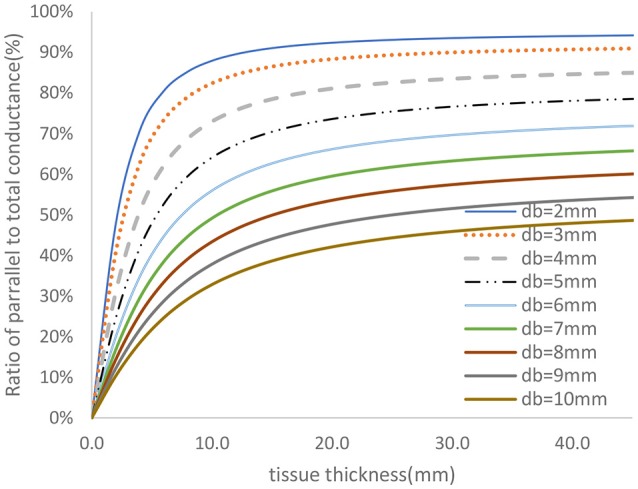
Ratio of parallel conductance to the total conductance for various lumen diameters for 5-5-5 guidewire based on model prediction. There is 0.45% saline solution in the lumen and 0.1% saline solution in the tissue which closely represent the *in-vivo* condition.

**Figure 5:**
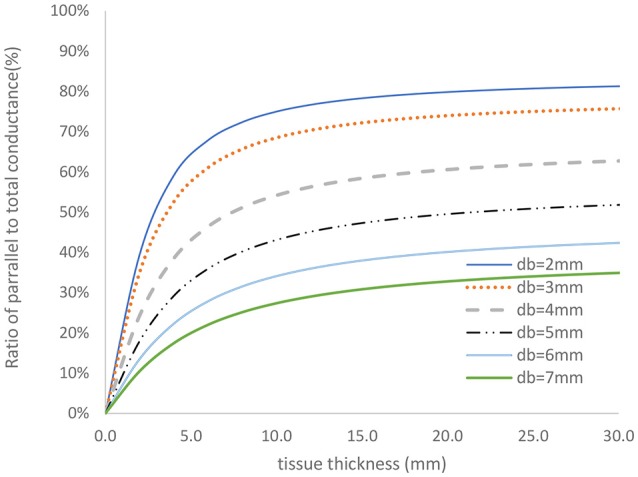
Ratio of parallel conductance to the total conductance for various lumen diameters for 2-2-2 guidewire based on model predication. There is 0.45% saline solution in the lumen and 0.1% saline solution in the tissue which closely represent the *in-vivo* condition.

The two-frequency approach was used to study the relative contribution of parallel conductance for various diameters and tissue thicknesses. The vessel wall conductance was accounted for as part of the parallel conductance. Figure 4 shows the ratio of the parallel conductance to the total conductance as function of tissue thickness for various diameters. The system consisted of 0.45% saline (similar conductivity as blood) solution in the lumen and 0.1% saline solution outside of the lumen (similar surrounding tissue conductivity to *in vivo*). The 0.45% solution conductivities were obtained in phantom tests as reported here. Most of the electrical conductance flows outside of the lumen at lower lumen diameters which makes the contribution of the lumen conductance to the total conductance negligible for the 5-5-5 guidewire. For the smaller vessels, the parallel conductance is reduced with the 2-2-2 guidewire as shown in Figure 5 where the excitation and the sensing lengths are smaller. The figure demonstrates the advantage of using 2-2-2 guidewire for the smaller vessels (2–4 mm in diameter) due to the lower ratio of the parallel conductance to the total conductance while the 5-5-5 guidewire is more appropriate for the larger vessels (4–10 mm in diameter) since they have lower parallel conductance and the electrical field can maintain cylindricity in the larger vessels (Choi et al., 2017).

**Figure 6:**
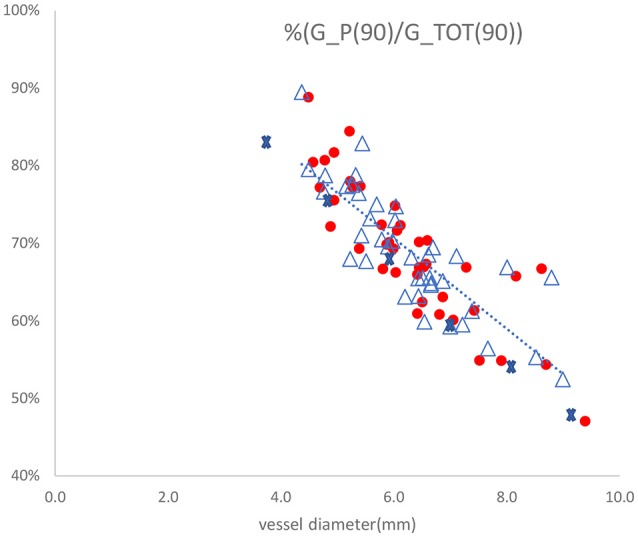
Ratio of parallel conductance to total conductance as function of vessel diameter for 25 patients with the two-injections method using 5-5-5 guidewire. The dotted line represents linear regression fit to the measured values with R-squared value of 0.682. The points shown by cross marks are the values predicted by the model. The other marks are the parallel conductance ratios measured twice for each patient with 0.9% saline solution.

One of the assumptions of the two-injections method is that the parallel conductance is the same when injecting two saline solutions at different electrical conductivities. The electrical conductivities of the two saline solutions differ by a factor of two. The parallel conductance model developed here can be used to validate the two-injections method assumption. This exercise has been applied to the clinical data (Nair et al., 2018) taken for 25 patients at different anatomical locations with the two-injections method and 5-5-5 guidewire. The vessel diameters vary between 4 and 9 mm. The ratio of parallel conductance to the total conductance as function of vessel diameter is shown in Figure 6 for the case of 0.9% saline solution. The total conductance in this figure has been measured with two measurements at each location. The parallel conductance was determined by subtracting the total conductance from the vessel conductance. The vessel conductance can be derived by the vessel diameter estimated from the two saline solution injections. It can be noted that this ratio drops with increase in diameters. The tissue conductivity value was estimated by equating the ratio of the parallel conductance to the total conductance in Figure 4 with the average ratio obtained in Figure 6 for only one diameter (e.g., 5 mm). The points shown by cross marks in Figure 6 are the model predictions. The results show that the assumption of equal parallel conductance can be justified as predicted by the model.

## Limitations of method

There are potential limitations to this approach that require further investigations. The two input parameters to the model, the surrounding tissue conductivity and the width or diameter of the surrounding tissue, are not known under *in vivo* tests and can only be estimated. The surrounding tissue conductivity can vary significantly; e.g., fat tissue conductivity is about 0.06 S/m at body temperature whereas heart muscle conductivity can be as high as 0.38 S/m at the same temperature (Gabriel, 1996). In reality, however, the variation is likely to be much less. The injection method can be used to determine the Gp value in patients in a particular vessel segment to assess variability as was done here. Although the width or diameter of the surrounding tissue can also vary, the variation is not as large. The width of the surrounding tissue of the peripheral vessels (limb) or coronary vessels (heart) can be physically estimated. The sensitivity analysis showed that the prediction is not sensitive to a reasonable variation in tissue thickness.

The limitation discussion above is more applicable to clinical translation than animal research. In the latter, the tissue conductivity and thickness can be determined invasively and hence measured directly (*in situ* or *ex-vivo*).

## Conclusion

A model was devised to estimate the lumen area and diameter of arteries without saline injections. Phantom and *ex-vivo* measurements analysis demonstrates that this technique is comparable to the injection method. This development has the potential to allow real-time pullback profiles of vessel lumen with accuracy to guide therapy delivery for treatment of vascular disease.

## Disclosures

Dr. Kassab is founder of 3DT Holdings.

## Author contributions

AD: Model development, experimental set-up, tests, data analysis, draft of the manuscript; GK: Critical review of the research direction and manuscript.

### Conflict of interest statement

The authors declare that the research was conducted in the absence of any commercial or financial relationships that could be construed as a potential conflict of interest.
