# Supplementary material for: Injection-Less Conductance Method for Vascular Sizing
Source: Front Physiol. 2018 Apr 12;9:371. doi: 10.3389/fphys.2018.00371 (PMC5906715; doi:10.3389/fphys.2018.00371)
Supplement: Supplementary file 1 [file DataSheet1.docx]

**Appendix**

***Mathematical modeling***

The electric field distribution in the lumen and the surrounding tissue is inhomogeneous which makes the conductance-diameter relationship nonlinear. The electric field intensity varies with distance from the center of the lumen. When the diameter increases, the far-field electric field contribution to the sensing electrodes decreases and hence the measured conductance approaches a saturation value. On the other hand, if the radius of the lumen decreases, the electric field distribution tends to be more homogeneous, so the conductance-diameter relationship is more linear.

Since the electric field generated by source electrodes is not homogeneous and the electric field intensity inside the lumen is not constant, the blood conductance can be obtained by solving Laplace’s equation which results in the dependence of $G_{b} ( real conductance)$as a function of lumen diameter as follows(Wei,2004):

|  | $G_{b}=\frac{\pi\sigma_{bReal}d\left( d^{2}-L^{2} \right)}{4L}\left( \frac{2}{\sqrt{d_{GW}^{2}+d^{2}}}-\frac{2}{\sqrt{d_{b}^{2}+d^{2}}} \right)$ | (A1) |
| --- | --- | --- |

where $d$ is the excitation electrode separation distance, $L$ is the sensing electrode separation distance, $\sigma_{bReal}$is the blood conductivity, $d_{GW}$is the diameter of the guidewire, and $d_{b}$ is the lumen diameter. This equation assumes that the radius of the sensing electrodes is small enough that their influences on the electric field distribution are negligible. It is important to note that $\sigma_{bReal}$ defined as real conductivity can be obtained by equation (A1) since all the elements of equation (A1) can be measured to obtain $\sigma_{bReal}$. In the literature, the value of conductivity has been obtained from the following equation which is only valid for a homogeneous field distribution: For a homogeneous field distribution, equation (A1) can be simplified to the following equation:

$G_{b}=\frac{{\pi\sigma}_{bIdeal}\left( d_{b}^{2}-d_{GW}^{2} \right)}{4L}$ (A2)

|  |
| --- |

The conductivity of the equation (A2) is called ideal conductivity since it represents the ideal conductance equation which may be an approximation to reality. The ratio of real to ideal conductivities is defined as $\sigma_{bReal}/\sigma_{bIdeal}$ and it has been plotted as function of the lumen diameter in Figure A1 for the two different guidewires. It is clear from Figure A1 that the real conductivity is higher than the idealized one using the same value for the conductance and this difference increases with lumen diameter. In other words, if one wants to use equation (A2) for conductance, the measured real conductivity should be divided by the $\sigma_{bReal}/\sigma_{bIdeal}$ which is designated as Blood Conductivity Correction Factor (BCCF), to find the ideal conductivity.

This is also true for the vessel wall where both real and ideal conductivities are defined. The vessel wall conductance is the same form as equation (A1) but with different parameters as follows:

| $G_{t}=\frac{\pi\sigma_{tReal}d(d^{2}-L^{2})}{4L}\left( \frac{2}{\sqrt{d_{b}^{2}+d^{2}}}-\frac{2}{\sqrt{d_{t}^{2}+d^{2}}} \right)$ | (A3) |
| --- | --- |

The new parameters in this equation are $\sigma_{tReal}$ and $d_{t}$. The $\sigma_{tReal}$ is defined as tissue wall real conductivity and $d_{t}$ is the outside diameter of the vessel wall. The $d_{t} and d_{b}$ are related by the following equation:

$d_{t}$= $d_{b}$ + 2t (A4)

where t is the tissue thickness of the vessel wall. The ratio of the wall tissue thickness and lumen diameter is defined as $\mathrm{TR}$:

$\mathrm{TR}$= t/$d_{b}$ (A5)

$d_{t}$= $d_{b}( 1+2\mathrm{TR}$) (A6)

The ideal tissue conductivity, $\sigma_{tIdeal}$, is defined by the following equation:$G_{t}=\frac{{\pi\sigma_{tIdeal}}\left( d_{t}^{2}-d_{b}^{2} \right)}{4L}$ (A7)

If equation (A7) is used for the vessel wall tissue conductance calculation, ideal tissue conductivity should be used. This will be obtained by dividing real tissue conductivity by the vessel wall Tissue Conductivity Correction Factor (TCCF) which is a function of lumen diameter, guidewire diameter, and TR which can be obtained from Figure A2 adjusted for the value of TR. TCCF is defined as the ratio of real to ideal tissue conductivity.

Blood is considered a heterogeneous medium because of the erythrocytes in plasma. Often the electrical characteristics of suspensions of blood are modeled using the well-known three-element model(Zhao,1993). In this lumped model, one resistor represents the electrical resistance of plasma, while the effect of the cell membrane capacitance of the erythrocytes is modeled by a capacitor. Furthermore, another resistor represents the effect of the interior cell resistance of the erythrocytes. We need to measure the admittance in three frequencies to model blood. Our analysis shows that the results are similar in the frequency range of our interest if we replace it with two-frequency model where the effect of the interior cell resistance is neglected. We adopted the two-frequency model for the sake of simplicity. The behavior of blood is therefore modeled in the frequency domain by a RC circuit where $R$ represents the blood resistance and C represents the capacitance across the red blood cell membrane. Blood impedance at two different frequencies needs to be measured to derive the values of $R$ and C.

The blood electrical conductivity remains not only a function of frequency, but it is also a function of diameter as evidenced by the in vitro experimental results which will be discussed later. This is due to several phenomena that occur on the interface between the electrode and the blood layer which acts like a capacitor with charge transfer and polarization resistance(Basic of Electrochemical Impedance Spectroscopy, <https://www.gamry.com/application-notes/EIS/basics-of-electrochemical-impedance-spectroscopy/>). This phenomenon was also observed in the case where the electrolyte was NaCl solution where there is no effect of the red blood cell interior resistance and its membrane acts like a capacitor. This is due to the electrolyte ions motion which increases with frequency and thus results in increase of electrical conductivity ­­_­_(Anderson,1994).

A two-frequency model can be best represented by a classical parallel RC-circuit to calculate the blood electrical conductivity as function of frequency. The value of the resistance, $R$, and capacitance, $C$, are a function of impedance and frequency as follows:

$R=\left[ \frac{{(Z_{1}Z_{2}\omega_{2})}^{2}-{(Z_{1}Z_{2}\omega_{1})}^{2}}{{(Z_{2}\omega_{2})}^{2}-{(Z_{1}\omega_{1})}^{2}} \right]^{\frac{1}{2}}$ (A8)

$C=\left[ \frac{R^{2}-Z_{2}^{2}}{{(Z_{2}\omega_{2}R)}^{2}} \right]^{\frac{1}{2}}$ (A9)

where$\omega_{i}=2\cdot\pi\cdot f_{i}$, $Z_{1}$and $Z_{2}$ are the impedances at frequencies $f_{1}$and $f_{2}$, which are the ratio of the measured voltages to the applied current. In the two-frequency model, the value of $R$ represents the total resistance of the combined system which includes both the blood in the lumen and the vessel wall. In the two-frequency model, the inverse $R$ is the total conductance of system.

The goal of this study is to develop a model to predict the parallel conductance. The measured total conductance in an infinite medium,$G_{inf}$, between two sensing electrodes can be approximated by the following equation^14^.

$G_{inf, bath}=\frac{\pi\sigma_{\boldsymbol{bath}}d(d^{2}-L^{2})}{4L}\left( \frac{2}{\sqrt{d_{GW}^{2}+d^{2}}} \right)$ (A10)

The parallel conductance for an infinite medium can be obtained from this equation by replacing $d_{b}$for $d_{GW}$ since anything beyond the lumen diameter is parallel conductance. The ratio of the parallel conductance for an infinite tissue thickness to the conductance inside of the lumen, is plotted in Figure A3 (in blue color) as function of lumen diameter for 2-2-2 guidewire, assuming that both the blood conductivity and the bath conductivity are the same. This graph demonstrates that the conductance inside the lumen is much smaller than the parallel conductance. Two factors are helpful to increase the share of the lumen conductance. One is that the blood conductivity is about 4-5 larger than the surrounding tissue conductivity and second is that the surrounding thickness is finite. Figure A3 also shows the ratio with blood conductivity of 0.7 S/m (in green color), where the lumen conductance contribution is much larger. For a finite surrounding tissue thickness, the parallel conductance is a function of surrounding tissue conductivity, electrical field distribution across the tissue and its thickness. The following model is proposed for the parallel conductance, $G_{bath},$ for a finite thickness of the surrounding tissue:

$G_{bath}=\frac{\pi\sigma_{bath}\cdot d(d^{2}-L^{2})}{4L}\left( \frac{2}{\sqrt{d_{t}^{2}+d^{2}}}-\frac{2}{\sqrt{d_{bath}^{2}+d^{2}}} \right)$ (A11)

where $\sigma_{bath} is the surrounding tissue conductivity and d_{bath}$ is the surrounding tissue thickness. An estimate of the surrounding tissue thickness is sufficient for the accurate determination of the parallel conductance since the electrical field drops rather abruptly from the center of the lumen and it is even less sensitive for the larger diameters of the lumen which is the region of interest for peripheral vessels. The tests results and comparison with the model are discussed in the result section.

**Appendix Tables**

**Table A1**. Measured voltages, mV, as function of phantom diameters and frequencies

for .45% saline solution at room temperature for 5-5-5 guidewire.

| **Saline 0.45%** |  |  |  |  |  |  |  |
| --- | --- | --- | --- | --- | --- | --- | --- |
| Voltage (mV) | **Diameter (mm)** | | |  |  |  |  |
| **(mV)** | 1.75 | 3 | 4 | 6 | 8 | (Nominal Dia (mm)) | |
|  | 1.75 | 2.97 | 3.96 | 6.00 | 8.00 | (Actual Dia (mm)) | |
| Freq (kHz) | 1.5074 | 2.83 | 3.86 | 5.93 | 7.95 | (Effective Dia (mm)) | |
| 1 | 259.00 | 61.00 | 55.00 | 25.60 | 15.60 |  |  |
| 10 | 209.00 | 57.00 | 52.00 | 24.30 | 14.90 |  |  |
| 20 | 176.00 | 55.00 | 48.00 | 23.50 | 14.50 |  |  |
| 40 | 125.00 | 50.00 | 43.00 | 21.70 | 14.00 |  |  |
| 60 | 90.00 | 44.00 | 38.00 | 19.40 | 13.00 |  |  |
| 80 | 69.00 | 39.00 | 33.00 | 17.50 | 12.00 |  |  |
| 100 | 55.00 | 34.00 | 30.00 | 16.00 | 11.00 |  |  |

**Table A2.** An example of R and C values for a 4 mm lumen diameter with 0.45% saline solution at room temperature for 5-5-5 guidewire. The frequency range is 1-80 KHz. Volt1 and volt2 are measured voltages at the corresponding frequencies. db is the calculated diameter.

| freq1(Hz) | freq2(Hz) | volt1(mV) | volt2(mV) | R(ohm) | C(F) | db(mm) |
| --- | --- | --- | --- | --- | --- | --- |
| 1,000 | 10,000 | 55 | 52 | 5.50E+02 | 1.00E-08 | 4.0056 |
| 1,000 | 20,000 | 55 | 48 | 5.50E+02 | 8.10E-09 | 4.0060 |
| 1,000 | 40,000 | 55 | 43 | 5.50E+02 | 5.77E-09 | 4.0064 |
| 1,000 | 60,000 | 55 | 38 | 5.50E+02 | 5.05E-09 | 4.0064 |
| 1,000 | 80,000 | 55 | 33 | 5.50E+02 | 4.82E-09 | 4.0065 |
| 10,000 | 20,000 | 52 | 48 | 5.36E+02 | 7.36E-09 | 3.9750 |
| 10,000 | 40,000 | 52 | 43 | 5.28E+02 | 5.37E-09 | 4.0018 |
| 10,000 | 60,000 | 52 | 38 | 5.27E+02 | 4.83E-09 | 4.0076 |
| 10,000 | 80,000 | 52 | 33 | 5.26E+02 | 4.70E-09 | 4.0089 |
| 20,000 | 40,000 | 48 | 43 | 5.01E+02 | 4.75E-09 | 4.0049 |
| 20,000 | 60,000 | 48 | 38 | 4.99E+02 | 4.52E-09 | 4.0127 |
| 20,000 | 80,000 | 48 | 33 | 4.99E+02 | 4.52E-09 | 4.0128 |
| 40,000 | 60,000 | 43 | 38 | 4.88E+02 | 4.38E-09 | 3.9976 |
| 40,000 | 80,000 | 43 | 33 | 4.91E+02 | 4.46E-09 | 3.9875 |
| 60,000 | 80,000 | 38 | 33 | 4.99E+02 | 4.52E-09 | 3.9399 |

**Table A3**. Calculation of 0.45% saline solution ideal conductivity, S/m, for

various phantom diameters and frequency for 2-2-2 guidewire at room temperature.

| Conductivity | **Diameter (mm)** | | |  |
| --- | --- | --- | --- | --- |
| **(S/m)** |  |  |  |  |
| Freq (kHz) | 4.0 | 3.30 | 2.50 | 1.75 |
|  |  |  |  |  |
| 10 | 1.1300 | 1.4000 | 1.4400 | 1.9000 |
| 20 | 1.1800 | 1.4500 | 1.4800 | 1.9500 |
| 40 | 1.2100 | 1.5000 | 1.5000 | 1.9700 |
| 60 | 1.2200 | 1.6000 | 1.6000 | 2.0300 |
| 80 | 1.2300 | 1.7000 | 1.6500 | 2.0500 |

**Appendix Figure Legends**

**Figure A1**. Saline solution conductivity correction factor (BCCF) as function of lumen diameter for two different guidewire diameters. The BCCF is defined as the ratio of real to ideal conductivities of the blood.

**Figure A2**. Vessel wall tissue conductivity correction factor (TCCF) as function of lumen diameter (TR=0.4). The TCCF is defined as the ratio of real to ideal conductivities of the tissue.

**Figure A3**. The ratio of parallel conductance to the conductance inside the lumen for an infinite tissue thickness as function of lumen diameter. The difference in the plots are due to the difference of conductivity inside the lumen.

***Figure A1*.** 0.45% saline solution conductivity correction factor (BCCF)

as function of lumen diameter for two different guidewire diameters.

BCCF is defined as the ratio of real to ideal conductivity of the blood.

**Figure A2**. Vessel wall tissue conductivity correction factor (TCCF) as

function of lumen diameter for 0.45% saline solution (TR=0.4).

TCCF is defined as the ratio of real to ideal conductivity of the tissue.

**Figure A3.** The ratio of parallel conductance to the conductance inside the

lumen for an infinite tissue thickness as function of lumen diameter.

The difference in the plots are due to the difference of conductivity inside

the lumen.

**Appendix References**

Anderson JE, The Debye-Falkenhagen effect: experimental fact or fiction. J of Non- Crystalline Solids, 172-174, Part 2, 1994.1190-1194.

Basic of Electrochemical Impedance Spectroscopy, Gamry Instruments, application-notes. <https://www.gamry.com/application-notes/EIS/basics-of-electrochemical-impedance-spectroscopy/>.

Wei CL. Improvements in the Accuracy of Estimation of Left Ventricular Volume from Measurements of Complex Admittance Using a Tetrapolar Catheter. Ph.D. Dissertation. The University of Texas at Austin. AAI3145878. 2004.Austin ,Texas.

Zhao T-X, Jacobson B, Ribbe T. Triple-frequency method for measuring blood impedance. [Physiological Measurement](http://iopscience.iop.org/journal/0967-3334), [Volume 14](http://iopscience.iop.org/volume/0967-3334/14), [Number 2](http://iopscience.iop.org/issue/0967-3334/14/2), 1993, 145-156.
